# The Relationship between Oral Health and Schizophrenia in Advanced Age—A Narrative Review in the Context of the Current Literature

**DOI:** 10.3390/jcm12206496

**Published:** 2023-10-12

**Authors:** Sanjana Santhosh Kumar, Raquel Cantillo, Dongxia Ye

**Affiliations:** Eastman Institute for Oral Health, University of Rochester Medical Center, Rochester, NY 14620, USA or sanjanasanthosh18@gmail.com (S.S.K.); or raquelcantillo197@hotmail.com (R.C.)

**Keywords:** schizophrenia, elderly, geriatric, antipsychotics, oral health

## Abstract

Schizophrenia is a psychiatric disorder that makes patients incompetent to perform day-to-day activities due to their progressing mental illness. In addition to disturbances with thoughts, behavioral changes, and impaired cognitive functions, oro-systemic health also becomes compromised. Even though the population with schizophrenia is primarily made up of older people, little is known about this group’s oral health treatment. The present review explores the relationship between oral healthcare and elderly patients with schizophrenia. Our literature search included databases, like PubMed, Embase, and Google Scholar, for appropriate and evidence-based information. Preventive and management strategies outlined in the included articles and future research perspectives in this field are discussed. To the best of our knowledge, this is the first review that looked at dental care and related characteristics in older schizophrenia patients. The findings highlight the necessity for targeted dental interventions to address the dental health challenges faced by this vulnerable population. Integrating dental health into the overall medical management of elderly individuals with schizophrenia is crucial. Although specific therapies remain limited, the emphasis is on preventive dentistry to reduce the occurrence and progression of oral diseases in this group.

## 1. Introduction

Schizophrenia is a chronic heterogenous psychological disorder whose symptoms include, but are not limited to, delusion and hallucination (positive symptoms), apathy, social withdrawal, anhedonia (negative symptoms), and cognitive deficits [1]. The description of schizophrenia has evolved through the years. The definition and boundaries have changed; however, its etiology and pathophysiology remain elusive. The International Classification of Diseases-11 (ICD-11) describes schizophrenia and other primary psychotic disorders by characteristics, such as significant impairments testing and alterations in behavior manifest in positive symptoms, such as persistent delusions, persistent hallucinations, disorganized thinking (typically manifest as disorganized speech), grossly disorganized behavior, and experiences of passivity and control, negative symptoms, such as blunted or flat affect and avolition, and psychomotor disturbances. The symptoms occur with sufficient frequency and intensity to deviate from expected cultural or subcultural norms. They do not arise as a feature of another mental and behavioral disorder (e.g., a mood disorder, delirium, or a disorder due to substance use). The categories in this grouping should not be used to classify the expression of ideas or beliefs, or be culturally sanctioned [2]. Most of the patient population continues to face challenges concerning occupational and social functioning [3]. Even though the population with schizophrenia is primarily made up of older people, little is known about this group’s oral health treatment. According to several authors, patients with schizophrenia do not always receive the best oral health treatment [4,5,6,7]. They are believed to be more vulnerable to developing dental diseases due to their decreased capacity for planning, lack of motivation, difficulty performing oral hygiene tasks, challenges they encounter by being refused treatment on the grounds of inability to maintain oral hygiene, and their increased propensity to experience adverse orofacial effects, such as xerostomia, sialorrhea, and oral dyskinesia brought on by psychotropic medications. Aside from age, one study found that gender, education, and minority status were not connected with dental care in older persons and were not relevant in schizophrenia [8]. On the other hand, due to the lengthy wait times and feelings of stigma, patients do not seek dental care with Medicaid, the public healthcare system in the United States [8]. To the best of our knowledge, this is the first review that looked at dental care and related characteristics in older schizophrenia patients. The aim of this narrative review is to provide an overview of the available literature on the correlation between geriatric patients diagnosed with schizophrenia and their oral health status.

## 2. Materials and Methods

**Figure 1:**
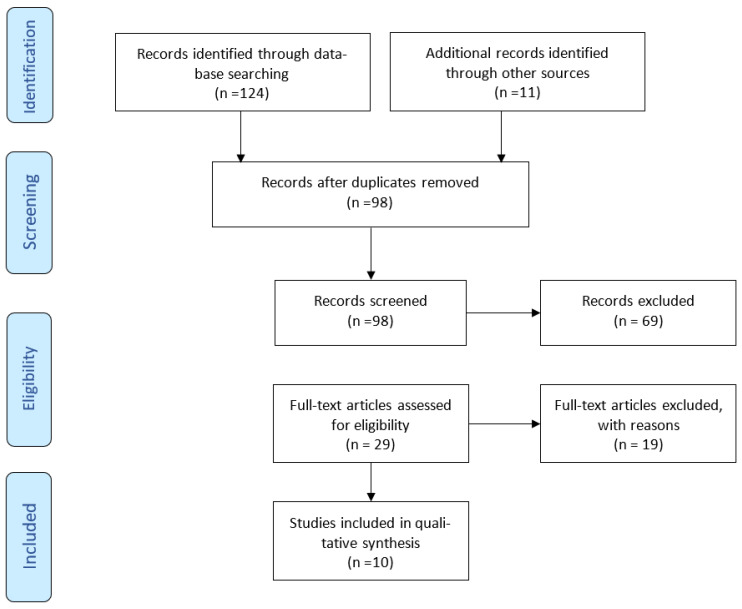
A flow diagram of the search strategy conducted (PRISMA flow of study selection process).

We conducted a literature review in the online electronic databases PubMed, Embase, and Google Scholar by searching for appropriate and evidence-based information. Our search strategy includes the keywords “Schizophrenia, Oral Care, Oral Health, Dental Care for Aged, Geriatric Dentistry, and Elderly”. Overall, 124 articles were found, and after removing duplicates and excluding articles based on our research question, 10 full-text articles were included in our review. The inclusion criteria were (i) original studies and (ii) schizophrenic patients older than 50 years old. Excluded were (i) review articles, (ii) schizophrenic patients less than 50 years old, and (iii) studies that categorized schizophrenic patients along with other mentally disordered patients in general (Figure 1).

## 3. Results

Table 1 discusses the literature showing oral health status in older adults with schizophrenia. It illustrates that older schizophrenia patients encounter higher oral health concerns, such as increased dental caries and poor periodontal health, as a result of medication-induced issues or inaccessibility to routine dental care. The table summarizes various studies conducted on the oral health of individuals with psychiatric disorders, particularly schizophrenia. Kurokawa et al. [9] conducted a cross-sectional study involving hospitalized patients with schizophrenia and found that older male patients with poor activities of daily living and low self-oral hygiene abilities exhibited poor oral hygiene. Joanna Ngo et al. [10] also conducted a cross-sectional study and discovered that male patients and older individuals had higher caries experience, with dry mouth being associated with typical antipsychotics. Zusman et al. [11] revealed in their cross-sectional study that the highest DMFT score was found in the older age group, emphasizing the need for improved dental care for chronic psychiatric patients. Denis et al. [12] conducted a qualitative study and highlighted the oral symptoms experienced by individuals with psychiatric disorders and the need for better oral hygiene support. Grinshpoon et al. [13] observed that patients taking atypical antipsychotics had better dental health than those on typical ones. Janarthanan et al. [9] emphasized the importance of targeting individuals with financial and cognitive difficulties for dental care. Chek Wey et al. [14] found poor oral hygiene and side effects of psychotropic medications among psychiatric patients, calling for preventive care. Tani et al. [15] revealed that older age, smoking, and less frequent tooth brushing were negatively related to dental conditions in schizophrenia patients. Kuan-Yu Chuet al. [16,17] identified various factors, including age, medication, and institutional stays, that influenced dental health in patients with schizophrenia. These studies collectively highlight the need for improved dental care and preventive strategies for individuals with schizophrenia, especially those who are older or facing challenges in their daily activities.

## 4. Discussion

### 4.1. Relationship between Etiology of Schizophrenia and Oral Health

Understanding and maintaining good dental health might be difficult for those with schizophrenia. There is evidence that mental health is connected to oral health and that they share a bidirectional relationship [18]. Dental problems frequently observed among psychiatric patients include periodontal disease and dental caries [19]. This is primarily because oral health is not emphasized in the setting of mental illness [20]. Periodontal disease and its progression may be negatively impacted by changes in inflammatory-related genes, genetic polymorphism of cytokines, and proinflammatory cytokine dysregulation in schizophrenia patients, which includes an elevation in levels of IL-1, IL-6, IL-9, TNF α, TNF β, PGE2, and CRP [21,22]. Mental disorders may also cause dysregulation of the hypothalamic–pituitary–adrenal and sympathoadrenal medullary axes. When these axes are dysregulated, a cascade of inflammatory, neurotransmitter, and hormonal mediators occurs [23]. As a result, an imbalance in the oral microbiome is created, causing decreased immunological function, altered salivary phenotype, and bruxism, all of which increase the likelihood of developing a variety of oral disorders, including dental caries, periodontal diseases, and tooth wear [24,25]. On the other hand, neuroinflammation in schizophrenic patients can also occur at advanced stages of periodontal diseases or oral abscesses when oral bacteria and/or their toxins invade the blood and/or cranial nerves [26].

### 4.2. Oral Manifestations

The aging process is linked to physical and sensory impairments causing inadequate oral health care. Adding the devastating effects of this psychiatric disorder has the potential to deteriorate their oral health further. Schizophrenia is characterized by impairing the subject’s capacity to carry out oral hygiene effectively, leading to a greater likelihood of experiencing poor oral health [6,13]. Research findings indicate that a significant proportion, ranging from 61% to 83% of individuals diagnosed with schizophrenia, experience dental disease [27]. The severity of the diagnosis, sex, social history, medications, and whether the patient is institutionalized or not play a role in oral health outcomes [6,9,10,13,28].

#### 4.2.1. Dental Caries

Most studies assess caries experience by utilizing DMFT scores. However, a limitation of this scoring system is that it not only reflects the impact of the disease but also considers the individual’s lifetime history of caries. Nevertheless, patients with schizophrenia have been associated with higher scores when compared to patients with other mental disorders [11]. A study conducted on 1108 institutionalized residents with schizophrenia concluded that age was a significant factor related to a high level of dental caries [16]. Other studies on this population have revealed that males tend to exhibit higher DMFT scores than females. This is attributed to factors, such as a history of smoking, being first-time visitors to a dental clinic, or negligence of dental care [9,10,28].

#### 4.2.2. Deficient Oral Hygiene

Individuals with schizophrenia often struggle with a lack of motivation, which can hinder their ability to maintain regular oral hygiene practices. These characteristics have been demonstrated in patients with severe negative symptoms and certain personality disorders [15,28]. Additionally, anxiety surrounding dental treatment may cause hesitation in seeking timely dental care. Consequently, this delay in seeking treatment can lead to the need for more complex and costly dental interventions or even losing dentition, further contributing to the individual’s anxiety. As previously mentioned, antipsychotic medications can impact fine motor skills. Tremors, in particular, can make it challenging for individuals to hold a toothbrush and properly perform flossing effectively. It has been found that a greater degree of tremor has been associated with a greater DMFT [15].

#### 4.2.3. Xerostomia

The use of antipsychotics and anticholinergics causes salivary gland hypofunction and xerostomia, which increases the incidence of dental caries. Due to experiencing dry mouth, individuals tend to consume sugary drinks more frequently, thereby exacerbating the chance of tooth decay [13,28,29,30]. The decrease in salivary flow among patients with schizophrenia results in a diminished buffering capacity, increasing the likelihood of an acidic oral environment promoting the demineralization of tooth enamel and increasing the rapid progression of decay [13,28,29,30].

#### 4.2.4. Effects of Tobacco

According to Winterer, individuals diagnosed with schizophrenia have a higher prevalence of smoking than patients with other mental disorders, with rates ranging from approximately 70% to 80%. It has been observed that smoking can have potential benefits for patients with schizophrenia, particularly in alleviating negative and cognitive symptoms [6,31]. This positive impact is believed to occur through the release of dopamine and stimulation of its activity, as well as the inhibition of its degradation [6,31]. Nevertheless, smoking has been positively associated with higher DMFT in several studies [13,28,29,30]. This fact has been consistent across samples in the literature. It is widely recognized that smoking not only contributes to an increase in pocket depths, loss of attachment, and loss of teeth affecting overall health, but it also increases the risk of developing oral cancer [15]. It is vital to promote smoking cessation programs in the dental office and perform oral cancer screenings routinely.

#### 4.2.5. Temporomandibular Disorders

Recent studies show that temporomandibular disorders are more prevalent after childbearing age (45–64) and gradually decrease as age advances. In older adults, TMD is self-limiting and can be treated [32]. Interestingly, in older patients with schizophrenia, these orofacial disorders are frequently observed due to the long-term use of antipsychotic medications and the comorbid conditions associated with the disease. These individuals may exhibit reduced sensitivity to somatic pain, resulting in delayed consultation and further joint deterioration [33]. The combination of masticatory muscle tension, clenching, grinding, and emotional distress places them at a heightened risk of developing temporomandibular disorders (TMD). A study by Gurbuz et al. reported a prevalence of any sign of TMD in 87% of patients with schizophrenia, and 39.2% of these patients exhibited severe tooth wear [33]. The reported percentages highlight a potential connection between these two conditions, raising the need for dental providers to assess and manage TMD, ensuring comprehensive oral care for this population.

#### 4.2.6. Tardive Dyskinesia

This adverse effect of antipsychotics causes involuntary movements of the extremities, trunk, and orofacial movements manifested as lip smacking, tongue protrusion, grinding mandibular movements, and puckering. These involuntary mandible movements can affect teeth and occlusion [13,34]. Individuals aged 60 and above are particularly vulnerable to the effects of medications. The progression of the disease is influenced by factors, such as the dosage and potency of the antipsychotics [34].

### 4.3. Dental Management

Dental treatment becomes difficult in this population due to several factors. There needs to be more communication between the dental provider and the patient. Cognitive impairments and disorganized thinking might hinder effective communication during dental visits, making understanding and addressing patients’ needs challenging. Having a caregiver present during dental visits can indeed be beneficial in managing the challenges associated with treating individuals with schizophrenia. Caregivers can provide support, help with communication, and assist in addressing the specific needs and concerns of the patient. They can also help promote oral hygiene practices and ensure adherence to dental care recommendations outside the dental office. The presence of a caregiver can enhance the whole dental experience for the patient and facilitate effective communication and cooperation between the dentist, patient, and caregiver.

Dental anxiety, agitation, or aggression is frequent in these patients. In some cases, dental procedures must be performed under nitrous oxide or sedation, which require special training, making the procedure more expensive for the patient. This limits the availability of specialized dental services for these individuals or a dentist willing to see them.

A study conducted by Zusman et al., reported that they observed a high number of extracted teeth (19 per patient, with one-third of the patients missing 26–32 teeth) due to caries and failure to restore them. They encourage dentists to work on preventive strategies to avoid extractions or complex procedures to preserve the natural dentition in this population [11].

The incorporation of fluoride applications during routine dental visits and prescriptions of high-fluoride toothpaste aid in countering the heightened susceptibility to dental caries often observed in this population. Complementing this, the utilization of specialized oral hygiene devices, tailored to accommodate potential motor and cognitive limitations [11], can facilitate effective plaque removal and maintenance of oral hygiene. Regular and frequent dental cleanings serve as a cornerstone of preventive care [11], enabling timely identification and management of dental issues. In addition, imparting nutrition education that emphasizes a reduction in sugar consumption [11] contributes significantly to oral health improvement [11]. Given the propensity for unhealthy dietary habits, such guidance can empower individuals with schizophrenia to make informed dietary choices that mitigate the risk of dental problems [11] These multifaceted preventive strategies, encompassing fluoride toothpaste, tailored oral hygiene devices, frequent cleanings, and nutritional education, collectively strive to address the intricate oral health needs of schizophrenia patients, enhancing their overall health outcomes.

According to a study by Grinshpoon et al., patients treated with atypical antipsychotics demonstrated better dental health than those taking typical antipsychotics [13]. This improvement is attributed to the reduced extrapyramidal effects associated with atypical antipsychotics, facilitating better medication adherence. As a result, symptom management is enhanced, leading to an improved quality of life and increased adherence to oral health regimes resulting in better oral health, less anxiety, and adequate dental treatment sought promptly [13]. Psychiatrists must consider the patient’s oral health when prescribing antipsychotics. Up-to-date guidelines must be available to all physicians concerning the appropriate use of these medications. It is critical to find the lowest possible dosage to maintain the patient’s fine motor function needed to brush and floss correctly, but most importantly, to reduce the overall adverse effects of these medications [15]. The prioritization of severe mental health symptoms over potential oral health issues may result in the under-recognition and undertreatment of dental diseases, such as xerostomia or oral cancer, in individuals with schizophrenia [35]. Because of this, dentists are encouraged to maintain an open communication channel and discussion with the physicians to inform the association of polypharmacy with xerostomia and dental diseases and the need to manage these conditions in a multi-disciplinary way [36].

## 5. Future Directions

Future progressive research is required to address the burden of oral illnesses and lessen the disparities that affect people with mental problems. E Joury, in their recent study, mentioned three examples of future research to improve oral health among people with mental disorders [18]. Regarding public health interventions, the research could evaluate the impact of water fluoridation or fluoride varnish on reducing oral health inequalities among schizophrenic patients. As far as health service interventions are concerned, designing financial models with incentives to include oral health as a part of mental health services would help deliver evidence-based oral health prevention and stabilization amongst these patients. Finally, social care interventions might include training caregivers on supporting oral healthcare routinely to these patients. An interdisciplinary team should conduct these in real-world settings, including all interventions [37]. Although these studies try to direct future research among people with any mental disorder, the same approach would be helpful for elderly schizophrenic patients.

## 6. Conclusions

Schizophrenia is a chronic heterogeneous psychological disorder that affects older adults. This study aimed to investigate the relationship between schizophrenia and dental health in the elderly population. The findings of our literature search underscore the need for targeted dental interventions to address the dental health challenges this vulnerable population faces. They highlight the importance of integrating dental health into the overall medical management of elderly individuals with schizophrenia. In terms of addressing the burden of oral diseases in elderly patients with schizophrenia, current dental research has been unable to develop efficient specific therapies. However, there is an emphasis on prioritizing preventive dentistry to minimize the occurrence and progression of dental conditions in this population.

## Figures and Tables

**Table 1:** Literature shows oral health status in older adults with schizophrenia.

Literature	Age (Years)	Participants	Measurements	Type of Study	Dental Challenge	Dental Manifestation	Dental Consideration	Conclusion
Kurokawa et al. [9]	63 (SD = 13)	249 hospitalized PWS	Oral hygiene (DMFT, calculus index, debris Index) and Revised Oral Assessment Guide. Related factors: hospitalization, chlorpromazine equivalents [CPZE], age, Barthel index [BI], frequency of cleaning teeth, and self-oral hygiene ability.	Cross-sectional	Positive correlation: Age and DMFT. Males have worse oral hygiene than females. High doses of antipsychotics associated with a decline in OH.	Poor oral hygiene. Being a male and having low activities of daily living were associated with poor oral hygiene. Advanced age associated with dental caries.	Male patients and patients with low ADL, advanced age, and inability to perform OH.	Older male patients with poor activities of daily living and low self-oral hygiene abilities have poor oral hygiene.
Joanna Ngo et al. [10]	24–80 (Age group: 60–80 = 64 patients)	191 inpatients (88.5% diagnosed with schizophrenia); 64 out of 191 subjects were elderly (age 60–80).	DMFT, salivary flow, soft tissue, gum inflammation, calculus. Medication, number of years since admission, dental visit status.	Cross-sectional	Dental caries experience > in older pts. Smokers were first-time visits. Most take antipsychotics with anticholinergics = dry mouth.	Males and older people had higher caries experience. Salivary gland hypofunction (SGH) associated with higher DMFT. Classical antipsychotics lower mean saliva flow and SGH. Pain (27.7%).	Pts needed scaling, OHI, dentures, extractions, mandibular dyskinesia, and restorations. Changing from reactive dentistry to preventive dentistry.	Poor OH. Males are at greater risk of dental caries. Pts are suffering from dry mouth, and typical antipsychotics and anticholinergics should be used with caution.
Zusman et al. [11]	53 (SD = 16)	254 patients; 64 out of 254 subjects were elderly (age 55–91).	Psychiatric diagnosis: majority 82.3% schizophrenia. Dental status: DMFT	Cross-sectional	The highest DMFT score was found in the 75–91 age group. Schizophrenia patients had the highest mean of carious and missing teeth.	Caries, missing teeth, high number of extractions due to caries/pain. Dentists are unwilling to invest in complex conservative or rehabilitative treatments because of the difficulty in treating psychiatric patients. High number of decayed and missing teeth.	Improving the oral health of the chronic psychotic patient. Dental treatment must be made available for these patients. More preventive and restorative care is needed. Dental services must be available in hospital/institution systems.	Need for prevention of dental disease and provision of dental treatment in long-term inpatients with psychiatric disease.
Denis et al. [12]	45.8 (SD = 9.5)	20 PWS	Experience of oral symptoms: dental and facial pain, oral dysfunctions, side effects of treatment. The stress created by dental care. Attitudes. Autonomy dimension in oral health.	Qualitative	Patient management. More extractions.	Dental pain, jaw pain, disfunctions: swallowing, chewing, talking. Dry mouth, burning sensation, taste of food changed due to medications, anxiety regarding treatment, trismus. Anticholinergic drugs on OHRQoL in older people and PWS. Side effects of antipsychotic drugs.	Poor oral health and OHRQoL. Support patients with daily oral hygiene.	PWS views and experiences are based on dimensions related to the experience of oral symptoms.
Grinshpoon et al. [13]	51.4 (SD = 14.5)	348 hospitalized patients from 14 psychiatric institutions in Israel.	DMFT index. Data on medication (typical or atypical antipsychotics).	Cross-sectional	Missing teeth and decayed teeth.	Medications: typical antipsychotics affect fine motor movements, decreasing the ability to brush. Both (typ/atyp) cause tardive dyskinesia (megative effect on teeth and occlusion). Both have anticholinergic side effects, causing xerostomia (caries). They take anticholinergics to reduce the extrapyramidal effects of typical antipsychotics.	Monotherapy with atypicals is superior to both atypical/typical. The choice is on the psychiatrist but the benefit of atypicals regarding dental health should be considered.	Patients taking atypicals have better dental health than patients on typical or with a combination of both.
Janarthanan et al. [8]	61.5 (SD = 5.6)	198 patients living in New York City.	Krause’s model of illness behavior in later life	Cross-sectional	<2 dental visits/year. Financial strain and lower executive cognitive function	“Denture or teeth problems”, higher levels of oral dyskinesia.	Targeting people with financial and cognitive problems to provide dental care.	Old schizophrenic adults did not receive two dental visits per year.
Chek Wey et al. [14]	54.8 (SD = 16)	543 PWS from a psychiatric institution.	DMFT, CPITN, and DI.	Cross-sectional	Poor oral hygiene, side effects of psychotropic medications, smoking and difficulty in optimal dental care (<access)	Increase in DMFT, gingival bleeding, and periodontal disease.	Preventive care, promotion of oral hygiene.	Levels of decay and periodontal disease were greatest in older patients due to neglect.
Tani et al. [15]	55.6 (SD = 13.4)	523 PWS from psychiatric hospitals in Japan.	DMFT, smoking status, daily intake of sweets, dry mouth, frequency of daily tooth brushing, and tremor.	Cross-sectional.	Smoking, tremor burden, and less frequent tooth brushing.	Increase in DMFT and increased risk of physical comorbidities.	Physicians be aware of aged smoking patients with schizophrenia and caregivers should be advised to encourage and help patients to perform tooth brushing more frequently.	Older age, smoking, severe tremor, and less frequent tooth brushing were negatively related with dental condition in patients with schizophrenia.
Kuan-Yu Chu et al. [16]	65+	122 PWS from psychiatric hospital in Taiwan.	DMFT, NRT, and care index.	Cross-sectional	Effects of FGA, older age, lower educational level, and longer hospital stays.	NRT being <24 and high levels of dental caries	No effects of FGA. Prolonged stay in institutions when decision-makers are planning for preventive strategies of oral health for institutionalized residents with schizophrenia	Older age had an independent effect on the risk of a high DMFT score.
Yu Chu et al. [17]	50.6 (SD = 10.88)	878 PWS from psychiatric hospital in Taiwan.	DMFT and BMI	Cross-sectional	Effects of SGA, older age and underweight.	Increase in DMFT	No effects of SGA. Psychologists and dentists should pay attention to the relationship between BMI and dental caries among hospitalized schizophrenic patients.	Older age and being underweight is a significant factor associated with the increased risk of dental caries in hospitalized patients with schizophrenia.

PWS—patients with schizophrenia; CI—calculus index; DI—debris index; DMFT—decay missing filled teeth; ROAG—Revised Oral Assessment Guide; CPITN—Community Periodontal Index and Treatment Needs; FGA—first-generation antipsychotics; SGA—second-generation antipsychotics, NRT—number of remaining teeth; BMI—body Mass Index; PWS—person with schizophrenia.

## Data Availability

Not applicable.
